# An attenuated lymphocytic choriomeningitis virus vector enhances tumor control in mice partly via IFN-I

**DOI:** 10.1172/JCI178945

**Published:** 2024-06-11

**Authors:** Young Rock Chung, Bakare Awakoaiye, Tanushree Dangi, Nahid Irani, Slim Fourati, Pablo Penaloza-MacMaster

**Affiliations:** 1 Department of Microbiology-Immunology, Feinberg School of Medicine, and; 2 Department of Medicine, Division of Allergy and Immunology, Feinberg School of Medicine and Center for Human Immunobiology, Northwestern University, Chicago, Illinois, USA.

**Keywords:** Immunology, Vaccines, Adaptive immunity, Cancer immunotherapy, Cellular immune response

## Abstract

Viral vectors are being used for the treatment of cancer. Yet, their efficacy varies among tumors and their use poses challenges in immunosuppressed patients, underscoring the need for alternatives. We report striking antitumoral effects by a nonlytic viral vector based on attenuated lymphocytic choriomeningitis virus (r3LCMV). We show in multiple tumor models that injection of tumor-bearing mice with this vector results in improved tumor control and survival. Importantly, r3LCMV improved tumor control in immunodeficient *Rag1^–/–^* mice and *MyD88^–/–^* mice, suggesting that multiple pathways contributed to the antitumoral effects. The antitumoral effects of r3LCMV were also observed when this vector was administered several weeks before tumor challenges, suggesting the induction of trained immunity. Single-cell RNA sequencing analyses, antibody blockade experiments, and knockout models revealed a critical role for host-intrinsic IFN-I in the antitumoral efficacy of r3LCMV vectors. Collectively, these data demonstrate potent antitumoral effects by r3LCMV vectors and unveil multiple mechanisms underlying their antitumoral efficacy.

## Abstract 2

The antitumoral effects of lymphocytic choriomeningitis virus vectors that extend to *Rag1^–/–^* and *MyD88^–/–^* mice suggest involvement of multiple mechanisms, including IFN-1 responses.

## Introduction

Cancer is linked to immunosuppression, which inhibits the ability of the immune system to clear tumor cells. A specific challenge in cancer immunotherapies is the presence of “cold tumors,” where the immune system fails to respond to tumor cells. This process can be triggered by the recruitment of Tregs to the tumor microenvironment, as well as by the upregulation of inhibitory receptors such as PD-1 and CTLA-4, among many other factors. While immune checkpoint therapy can partially reverse immunosuppression and result in effective tumor control, only approximately 30% of patients respond, highlighting the need for alternative immunotherapies. Viruses have emerged as attractive therapies to overcome immunosuppression during cancer. In particular, oncolytic viruses that preferentially infect and replicate in tumor cells have been extensively explored for cancer immunotherapy (1). Currently, an oncolytic virus (talimogene laherparepvec, T-VEC) is approved for melanoma patients (2). Although this vector can be effective in some patients with melanoma, adverse effects have been reported following the use of this replicating lytic virus, and not all patients respond. Owing to safety concerns, immunocompromised patients are typically excluded from receiving this replicating lytic viral therapy, motivating the development of alternative viral vectors for cancer immunotherapy.

In this study, we explored a nonlytic virus, lymphocytic choriomeningitis virus (LCMV), as a cancer immunotherapy. LCMV can be engineered to serve as a replication-attenuated vector that can deliver foreign antigens to the immune system (3, 4). Prior studies have shown that immunization of mice with attenuated LCMV vectors expressing tumor antigens improves tumor control, and there is an ongoing trial evaluating the efficacy of attenuated LCMV vectors expressing HPV antigens in patients with HPV16^+^ metastatic head and neck carcinoma (ClinicalTrials.gov NCT04180215) (5–7). The use of viral vectors expressing a cargo of tumor antigens requires knowledge of specific tumor antigens, which may differ depending on the patient and the type of tumor. In this study we interrogated whether replication-attenuated r3LCMV vectors that do not express any tumor antigen provide antitumor protection. Using multiple tumor models, we show that injection of tumor-bearing mice with r3LCMV vectors results in improved tumor control and prolonged survival. Moreover, we demonstrate that the antitumoral effects of r3LCMV are partly dependent on the IFN-I pathway.

## Results

### Comparative analyses of antitumoral effects by replicating and non-replicating LCMV vectors.

**Figure 1:**
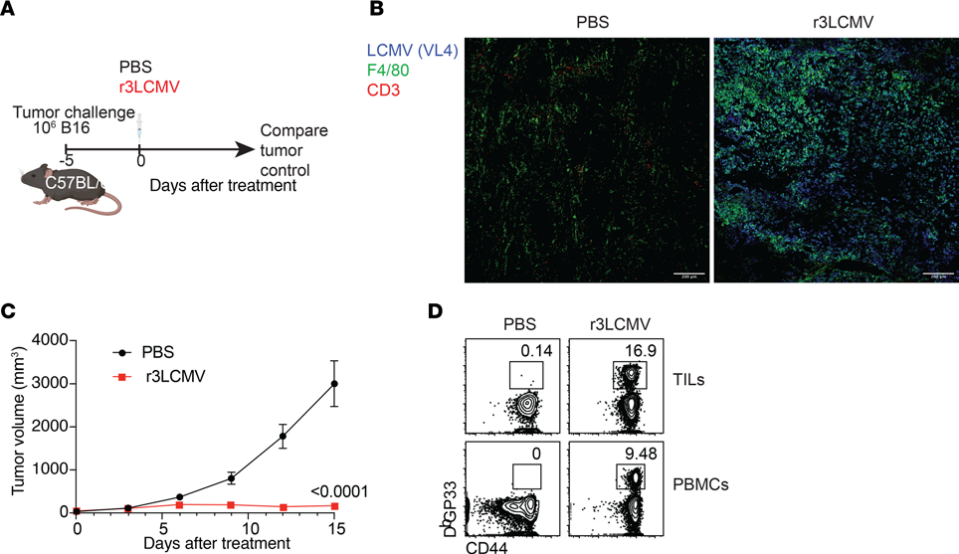
r3LCMV replicates in B16 tumors and improves tumor control. (**A**) Experiment outline for evaluating whether r3LCMV improves tumor control. (**B**) Representative immunofluorescence staining in tumor sections at day 4 after treatment. We used an LCMV nucleoprotein-specific antibody (clone VL4) to label virus-infected cells in tumor sections. Scale bars: 200 μm. (**C**) Tumor control. (**D**) Representative FACS plots showing LCMV-specific CD8^+^ T cell responses at day 7 after treatment (gated on live CD8^+^ T cells). TILs, tumor-infiltrating lymphocytes. Mice were treated intratumorally with 2 × 10^5^ focus-forming units (FFU) of r3LCMV, 5 days after subcutaneous tumor challenge. Before r3LCMV treatments, groups were distributed evenly according to tumor size. Data are pooled from 2 experiments (one experiment with *n* = 5 per group and another with *n* = 7 per group). Error bars represent SEM. Indicated *P* values were calculated by the Mann-Whitney test.

Because of their high immunogenicity, LCMV vectors have been explored as vaccine candidates for various diseases (8–10). In these prior studies, LCMV vectors have been genetically modified to include a foreign antigen to prime antigen-specific immune responses. In our study, however, we tested whether an LCMV vector that does not express any tumor antigen can confer “bystander” protection against tumor challenges in mice. We first challenged C57BL/6 mice with 10^6^ B16 melanoma cells, and at day 5 after challenge, we treated tumor-bearing mice intratumorally with 2 × 10^5^ focus-forming units of a replication-attenuated LCMV vector (r3LCMV) (Figure 1A). At day 4 after treatment, we harvested tumors and measured viral antigen by immunofluorescence. Viral antigen was highly colocalized with F4/80^+^ cells in mice treated with r3LCMV, suggesting that macrophages were preferentially infected with r3LCMV (Figure 1B). We also interrogated whether r3LCMV could replicate in B16 melanoma cells. To answer this question, we incubated B16 melanoma cells for 48 hours with r3LCMV vectors expressing a reporter green fluorescent protein (GFP) at a multiplicity of infection (MOI) of 0.1, and then we measured viral antigen by immunofluorescence. The r3LCMV vector was able to infect B16 melanoma cells in vitro, consistent with a prior study (11); for comparison, we included a non-replicating (rLCMV) vector, which was able to enter melanoma cells, but resulted in lower antigen levels (Supplemental Figure 1, A and B; supplemental material available online with this article; https://doi.org/10.1172/JCI178945DS1).

**Figure 2:**
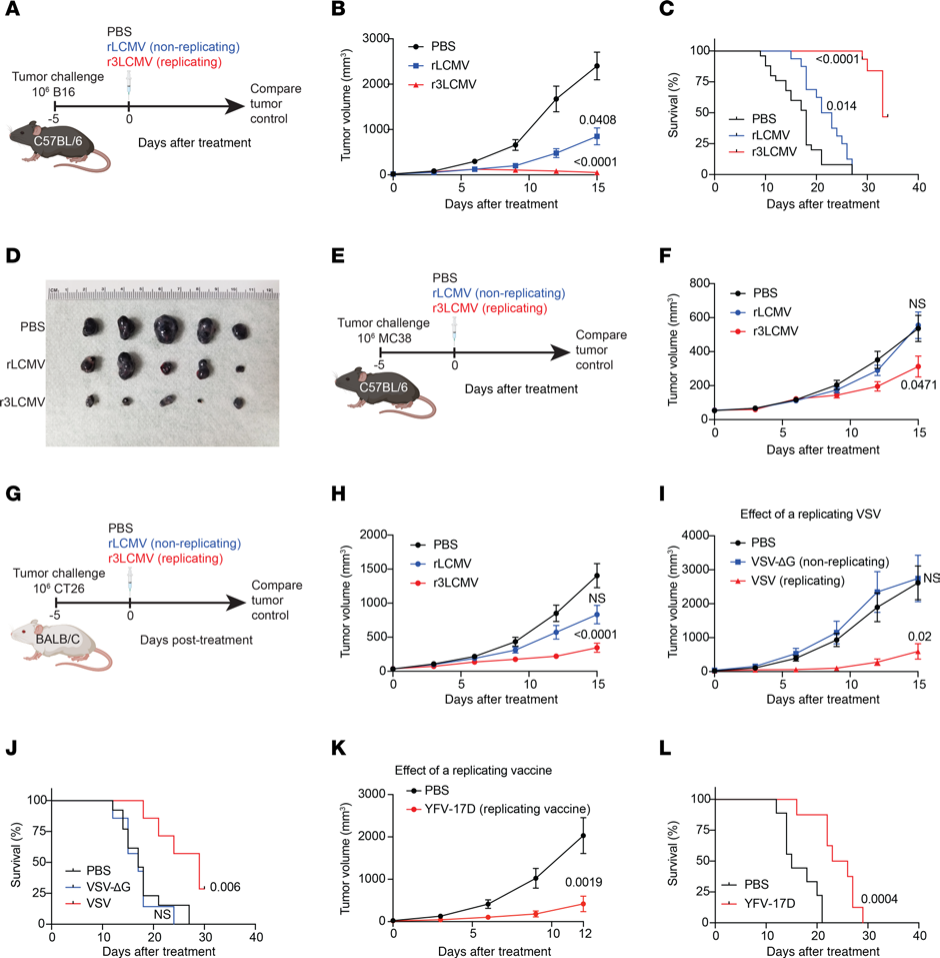
Comparing the antitumoral effects of replicating versus non-replicating viral vectors. (**A**–**D**) Effect of replicating (r3LCMV) versus non-replicating (rLCMV) vectors in the B16 melanoma model in C57BL/6 mice. (**A**) Experiment outline. The setup was similar to that in Figure 1 but comparing replicating versus non-replicating LCMV vectors. (**B**) Tumor control. (**C**) Survival. (**D**) Representative images of tumors at day 8 after treatment. (**E** and **F**) Effect of replicating versus non-replicating LCMV vectors in the colon adenocarcinoma model in C57BL/6 mice. (**E**) Experiment outline. (**F**) Tumor control. (**G** and **H**) Effect of replicating versus non-replicating LCMV vectors in the CT26 colon carcinoma model in BALB/c mice. (**G**) Experiment outline. (**H**) Tumor control. (**I** and **J**) Effect of replicating versus non-replicating VSV in the B16 melanoma model in C57BL/6 mice. (**I**) Tumor control. (**J**) Survival. (**K** and **L**) Effect of replicating YFV-17D vaccine in the B16 melanoma model in C57BL/6 mice. (**K**) Tumor control. (**L**) Survival. Mice were treated intratumorally with 2 × 10^5^ FFU of the indicated viruses, 5 days after tumor challenge. LCMV data are pooled from 2 experiments per tumor model (*n* = 5–13 per group). VSV data are pooled from 2 experiments per tumor model (*n* = 3–7 per group). YFV-17D data are pooled from 2 experiments per tumor model (*n* = 4–5 per group). Error bars represent SEM. Indicated *P* values in the tumor volume plots were calculated by the Mann-Whitney test, or by Kruskal-Wallis test with Dunn’s multiple-comparison test when comparing more than 2 groups. Indicated *P* values in the survival plots were calculated by the log rank test.

We also evaluated whether intratumoral treatment with r3LCMV improves tumor control. Interestingly, treatment of tumor-bearing mice with r3LCMV induced a significant improvement in tumor control (Figure 1C) associated with generation of virus-specific CD8^+^ T cell responses (Figure 1D). To determine whether the antitumoral effect of the LCMV vector was affected by the ability of the virus to replicate, we injected B16 melanoma–bearing mice with replicating or non-replicating LCMV vectors (both were attenuated relative to wild-type LCMV). We used the bisegmented rLCMV vector that can enter cells and express viral proteins but cannot induce a second round of infection because of a genetic absence of the glycoprotein (GP) gene, which encodes the viral protein that mediates viral entry. During the in vitro production of this bisegmented rLCMV vector, the GP is only provided in *trans* in the producer cells to allow entry of the vector into host cells, but progeny virions are unable to form infectious progeny virions owing to genetic lack of the GP. On the other hand, the r3LCMV vector expresses GP in *cis*, allowing it to undergo several replication cycles until it is eliminated by the host’s immune response, but it is still significantly attenuated and does not replicate to wild-type levels (12) (Supplemental Figure 1C). Interestingly, the replicating (r3LCMV) vector resulted in a superior antitumoral effect relative to the non-replicating (rLCMV) vector (Figure 2, A–D).

Intratumoral r3LCMV therapy also induced potent antitumoral effects in other tumor models, such as the MC38 colon adenocarcinoma (Figure 2, E and F), and in mice with different genetic backgrounds (Figure 2, G and H), suggesting a generalizable antitumor effect independent of the major histocompatibility complex (MHC) haplotype. To a lesser extent, a replicating vesicular stomatitis virus (VSV) and the replicating yellow fever virus (YFV-17D) vaccine also induced antitumoral effects (Figure 2, I–L), suggesting that replicating viral vectors exert superior antitumoral effects compared with non-replicating viral vectors. We also tested antitumoral effects on distal tumors, known as abscopal effects. To test this, we injected both flanks of the mice with B16 melanoma cells, followed by intratumoral r3LCMV injection in the right tumor only. Interestingly, r3LCMV also induced partial regression of the contralateral (left) tumor (Supplemental Figure 2), demonstrating the induction of abscopal effect. Further, we compared tumor control elicited by r3LCMV versus PD-L1 blockade (Supplemental Figure 3A). Treatment with r3LCMV alone resulted in significantly superior antitumoral control relative to PD-L1 blockade alone, and there was a pattern of improved survival in mice that received combined treatment, although the difference was not statistically significant (Supplemental Figure 3, B and C).

In addition, we observed that treatment of tumor-bearing mice with r3LCMV induced a significant reduction of systemic and tumor-draining lymph node Tregs after a week of treatment (Supplemental Figure 4, A–C). Depletion of Tregs has been shown to improve tumor control (13), so we examined whether Treg depletion could synergize with r3LCMV treatment. We used FoxP3-DTR mice, which allow for depletion of Tregs upon diphtheria toxin administration (14). Our data show that Treg depletion combined with r3LCMV treatment resulted in more potent antitumoral control relative to Treg depletion alone (Supplemental Figure 4, D and E).

### Role for antigen presentation, costimulation, and T cells.

**Figure 3:**
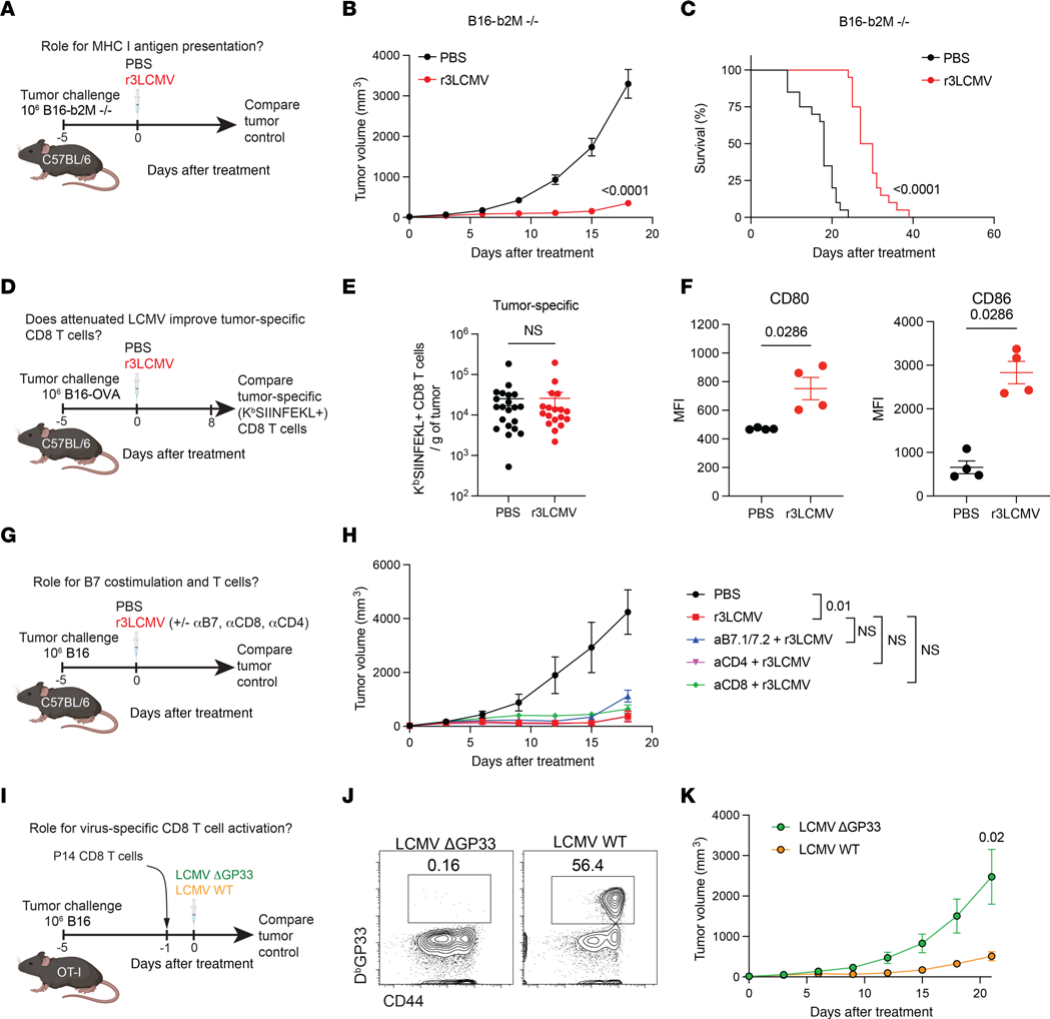
r3LCMV exerts antitumoral effects independent of CD8^+^ T cells and B7/CD28 costimulation. (**A**–**C**) Effect of r3LCMV vectors in the B16-*B2m^–/–^* melanoma model. (**A**) Experiment outline for evaluating the role of MHC I. (**B**) Tumor control. (**C**) Survival. (**D** and **E**) Effect of LCMV vectors on tumor-specific CD8^+^ T cell responses. (**D**) Experiment outline for measuring tumor-specific CD8^+^ T cells in the tumor. (**E**) Tumor-specific CD8^+^ T cells at day 8 after treatment. (**F** and **G**) Upregulation of B7 costimulatory molecules by r3LCMV. (**F**) CD80 and CD86 costimulatory molecules on dendritic cells from tumor-draining lymph nodes. Dendritic cells were gated on live, CD3^–^, NK1.1^–^, Ly-6G^–^, CD19^–^, CD11b^+^, CD11c^+^ at day 4 after treatment. (**G** and **H**) Effect of B7 costimulation blockade, CD8^+^ T cell depletion, and CD4^+^ T cell depletion. B7.1/B7.2-blocking antibodies, CD8^+^ T cell–depleting antibodies, or CD4^+^ T cell–depleting antibodies were administered i.p. every 3 days (see Methods for dosing information). (**G**) Experiment outline for evaluating the role of T cells and costimulation. (**H**) Tumor control. (**I**–**K**) Effect of virus-specific CD8^+^ T cells. (**I**) Experiment outline for evaluating the role of virus-specific T cell activation. (**J**) Representative FACS plots showing P14 cell expansion in PBMCs at day 7 after treatment. (**K**) Tumor control. Data from **A**–**C** are pooled from 2 experiments (one experiment with *n* = 10 per group and another with *n* = 10 per group). Data from **D** and **E** are pooled from 2 experiments (one experiment with *n* = 9 per group and another with *n* = 9–12 per group). Data from **F** are from 1 representative experiment (*n* = 4 per group). Data from **G** and **H** are from 1 representative experiment (*n* = 6–7 per group). Data from **I**–**K** are from 1 representative experiment (*n* = 6–7 per group). Error bars represent SEM. Indicated *P* values in the tumor volume plots were calculated by the Mann-Whitney test, or by Kruskal-Wallis test with Dunn’s multiple-comparison test when comparing more than 2 groups. Indicated *P* values in the survival plot were calculated by the log rank test.

T cells are thought to be critical for the control of tumors, and their activation is dependent on MHC antigen presentation and costimulation. We first examined the role of antigen presentation by challenging mice with β_2_-microglobulin–KO (*B2m^–/–^*) B16 melanoma cells, which are unable to present antigen to CD8^+^ T cells (15). *B2m^–/–^* B16 tumor–bearing mice were then treated with PBS or r3LCMV, and tumor control was measured. Interestingly, *B2m^–/–^* B16 tumor–bearing mice treated with r3LCMV showed improved tumor control relative to control-treated mice, suggesting that antigen presentation via MHC was not completely required for the antitumoral effect (Figure 3, A–C).

We then evaluated whether the r3LCMV treatment improves tumor-specific T cell responses, by challenging mice with B16 melanoma cells expressing ovalbumin (B16-OVA) and then measuring OVA-specific CD8^+^ T cell responses by K^b^SIINFEKL tetramer staining. The r3LCMV treatment did not improve tumor-specific (SIINFEKL-specific) CD8^+^ T cell responses (Figure 3, D and E). We then measured costimulatory molecule expression on dendritic cells (DCs) from tumor-draining lymph nodes following r3LCMV treatment, and we observed a significant increase in CD80 (B7.1) and CD86 (B7.2) molecule expression in mice that received r3LCMV treatment (Figure 3F), suggesting a role for B7 costimulation. However, blockade of B7.1 and B7.2 molecules did not abrogate the antitumoral effect of r3LCMV (Figure 3, G and H), suggesting that B7/CD28 costimulation was dispensable for the antitumoral effect of r3LCMV. We further examined the role of CD4^+^ T cell responses by depleting these cells using depleting antibodies. CD4^+^ T cell depletion did not impair the antitumoral effect of r3LCMV (Figure 3H).

Moreover, we performed CD8^+^ T cell depletion experiments to evaluate whether CD8^+^ T cells were mechanistically involved. CD8^+^ T cell depletion did not significantly impact the antitumoral effect of r3LCMV (Figure 3H). These findings did not necessarily indicate that T cells are dispensable for the antitumoral effect of r3LCMV, since treatment with T cell–depleting antibodies may not fully deplete all T cells in tissues. Thus, we used an adoptive CD8^+^ T cell transfer model to more rigorously measure the contribution of virus-specific CD8^+^ T cells in tumor control. We adoptively transferred TCR-transgenic CD8^+^ T cells recognizing the LCMV GP33-41 epitope (P14 cells) into recipient tumor-bearing OT-I mice, which contain only OVA-specific CD8^+^ T cells. This adoptive transfer model allowed us to examine the contribution of virus-specific CD8^+^ T cell activation in a “T cell–replete” environment. We used OT-I mice as recipients instead of *Rag1^–/–^* mice because transferring donor T cells into *Rag1^–/–^* mice would lead to rapid homeostatic proliferation of donor T cells (emptiness-induced proliferation) and other immune abnormalities caused by the absence of T cells and B cells (16). One day after P14 cell transfer, recipient mice were infected intratumorally with an LCMV variant lacking the GP33-41 epitope (LCMV ΔGP33) or a wild-type LCMV (Cl-13) to determine whether the activation of virus-specific CD8^+^ T cells potentiates tumor control (Figure 3I). Both LCMV strains replicate at comparable levels, and the only difference is a valine to alanine (V→A) escape mutation that destroys the GP33 epitope recognized by P14 cells (17). As expected, intratumoral treatment with the wild-type LCMV (but not the LCMV ΔGP33 variant) triggered robust P14 cell expansion in the tumor-bearing mice (Figure 3J). Interestingly, the mice that were infected with the wild-type LCMV (which showed robust P14 expansion) exhibited superior tumor control relative to the mice that were infected with the LCMV ΔGP33 variant (Figure 3K), suggesting that “bystander” activation of virus-specific CD8^+^ T cells can facilitate tumor control in a host devoid of tumor-specific T cell responses. Collectively, these data using transgenic P14 cells suggested that the bystander activation of virus-specific T cells could potentiate tumor control, without the need for tumor-specific T cells.

### r3LCMV improves tumor control in the absence of adaptive immunity.

**Figure 4:**
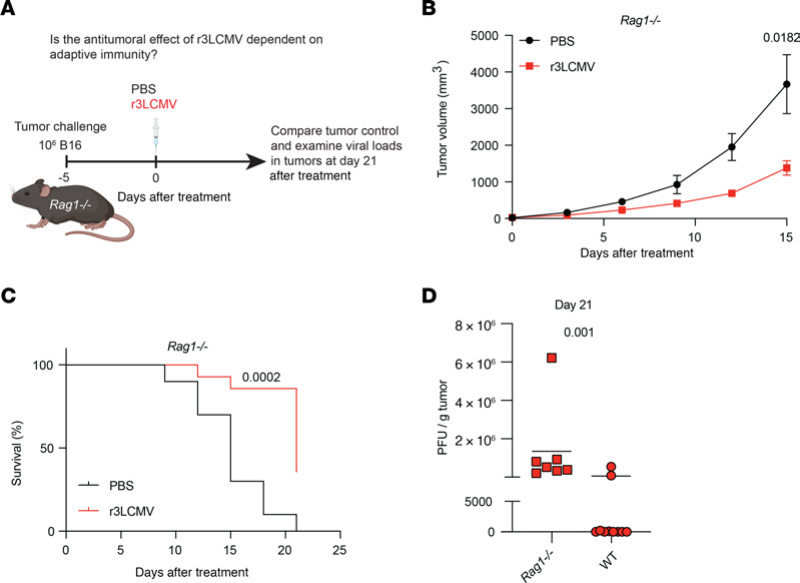
r3LCMV therapy improves tumor control in *Rag1^–/–^* mice. (**A**) Experiment outline. (**B**) Tumor control. (**C**) Survival. (**D**) Viral loads in tumors at day 21 after treatment. Viral loads were quantified by plaque assays on Vero cell monolayers. Data from **B** and **C** are pooled from 2 experiments (one experiment with *n* = 5 per group and another with *n* = 5–9 per group). Data from **D** are from the tumors of 7 *Rag1^–/–^* mice that were treated with r3LCMV and survived until day 21 (tumors of 10 wild-type mice that were treated with r3LCMV and survived until day 21 are included for comparison). Error bars represent SEM. Indicated *P* values were calculated by the Mann-Whitney test, or log rank test when comparing survival.

To interrogate the role of adaptive immunity more rigorously, we challenged *Rag1^–/–^* mice with B16 tumor cells, followed by treatment with r3LCMV (Figure 4A). *Rag1^–/–^* mice are unable to generate mature T cells and B cells, leading to severe combined immunodeficiency. Interestingly, *Rag1^–/–^* mice also exhibited a significant improvement in tumor control after r3LCMV treatment, demonstrating that r3LCMV could also induce antitumoral effects in the absence of adaptive immunity (Figure 4, B and C). These data do not necessarily contradict our findings above using P14 cells. We reason that although virus-specific T cells can facilitate tumor control, they are not the only component of the immune response that is required for the antitumoral effect of r3LCMV. It is also important to mention that the surviving *Rag1^–/–^* mice that were treated with r3LCMV were unable to clear the vector owing to lack of T cells (Figure 4D), but they appeared normal and without any signs of disease. We also examined whether the antitumoral effect of r3LCMV was dependent on IFN-γ, also known as “adaptive interferon.” IFN-γ is expressed mostly by effector T cells, and this cytokine is important for tumor control (18). To examine the role of IFN-γ on tumor cells, we challenged C57BL/6 mice with B16 melanoma cells lacking the IFN-γ receptor (*Ifngr1^–/–^*). We then treated mice with PBS or r3LCMV to examine whether tumor control by r3LCMV therapy was dependent on tumor-intrinsic IFN-γ signaling. Importantly, tumor control by r3LCMV was not dependent on tumor-intrinsic IFN-γ signaling (Supplemental Figure 5), suggesting that tumor control by r3LCMV therapy was not dependent on tumor-intrinsic “adaptive” interferon signaling.

### Single-cell RNA sequencing analyses reveal a role for type I interferons.

**Figure 5:**
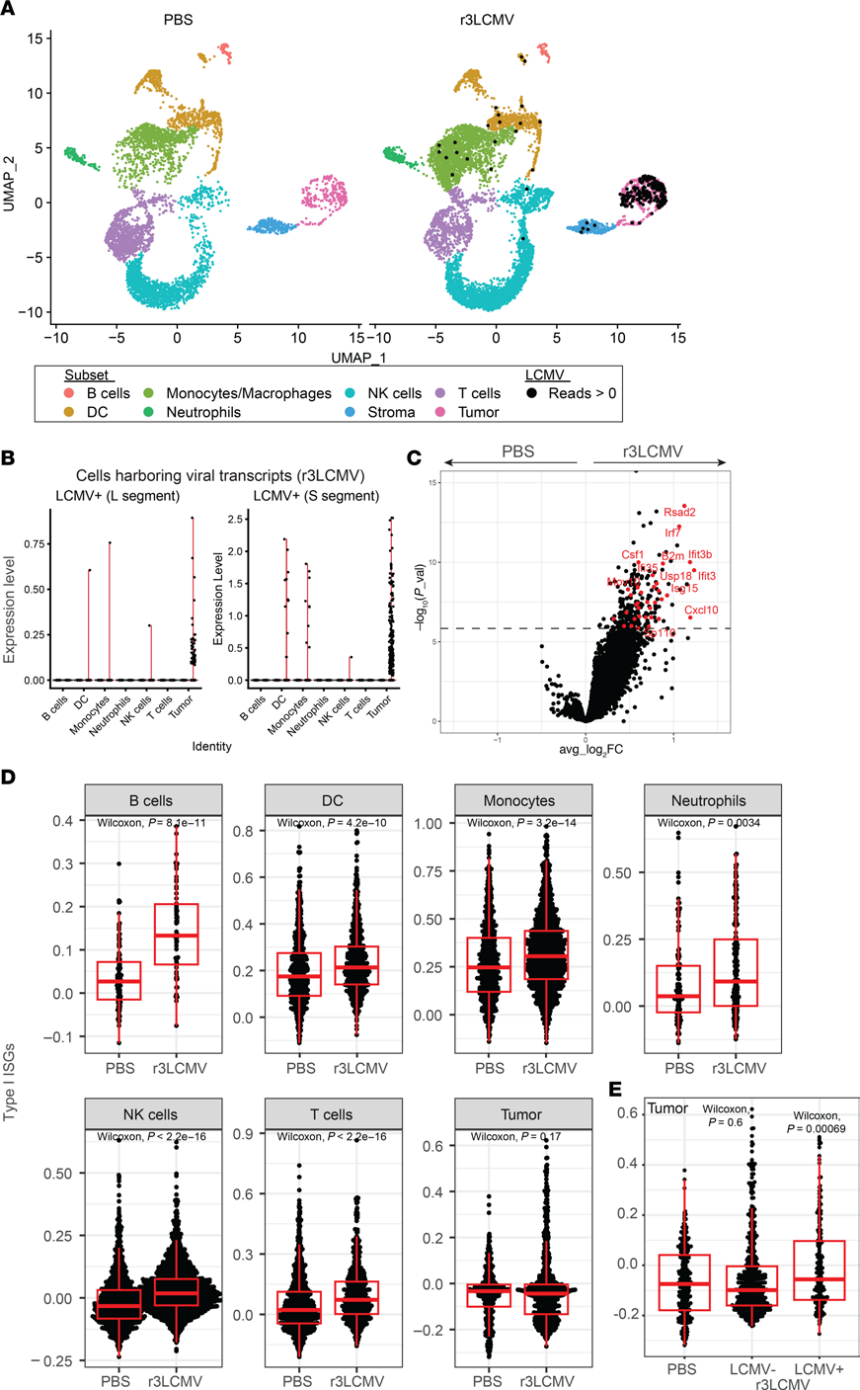
scRNA-Seq reveals enrichment of IFN-I responses by r3LCMV therapy. We performed gene expression analyses to understand the effects of r3LCMV on different cell subsets within the tumor microenvironment at day 4 after treatment. (**A**) Uniform manifold approximation and projection (UMAP) plots showing cell distribution based on RNA expression. Each cell is colored by its inferred subset (based on the Immunological Genome Project [ImmGen] database). Cells harboring LCMV reads are indicated by a black dot. (**B**) Level of expression of LCMV L and S transcripts on different cell subsets from r3LCMV-treated mice. (**C**) Volcano plot showing the differential expression of genes in tumor cells harboring LCMV reads versus those without LCMV reads. The dashed line indicates *P* value adjusted for multiple testing of 0.05. ISGs are indicated in red. (**D**) Enrichment for ISGs in different cell subsets. (**E**) ISGs on tumor cells harboring LCMV or not harboring LCMV. This panel shows that tumor cells with LCMV reads express higher levels of ISGs relative to tumor cells without LCMV reads. For each box plot, the vertical line indicates the median, the box indicates the interquartile range, and the whiskers indicate 1.5 times the interquartile range. Approximately 80% of cells were CD45^+^ (after MACS purification). Each group represents pooled tumors from 5 different mice. Indicated *P* values were calculated by Wilcoxon’s test.

We then performed gene expression analyses to understand the effects of r3LCMV on different cell subsets within the tumor microenvironment. We harvested tumors at day 4 after treatment, followed by single-cell RNA sequencing (scRNA-Seq) analyses. We observed differences in cell populations between the PBS- and r3LCMV-treated mice. Our single-cell gene expression data show that r3LCMV induced changes in cell frequencies within the tumor microenvironment, including a significant influx of natural killer (NK) cells and macrophages (Figure 5A). LCMV viral reads were detected in r3LCMV-treated mice, especially in dendritic cells, macrophages, and tumor cells themselves (which harbored the L and S RNA segments from LCMV) (Figure 5, A and B). These gene expression data also show that r3LCMV induced several IFN-induced genes (ISGs), including those coding for the master transcription factor Irf7 and transcripts for the antiviral proteins Ifi3 and Isg15 (Figure 5C). ISGs were significantly upregulated in immune cell subsets, but not in tumor cells when analyzed as a whole (when compounding uninfected and infected tumor cells) (Figure 5D). However, when we compared tumor cells containing viral transcripts with tumor cells lacking viral transcripts in the r3LCMV-treated mice, we observed significant upregulation of ISGs only in tumor cells containing viral transcripts (Figure 5E). These data suggested a possible role for IFN-I in the antitumoral control elicited by r3LCMV.

**Figure 6:**
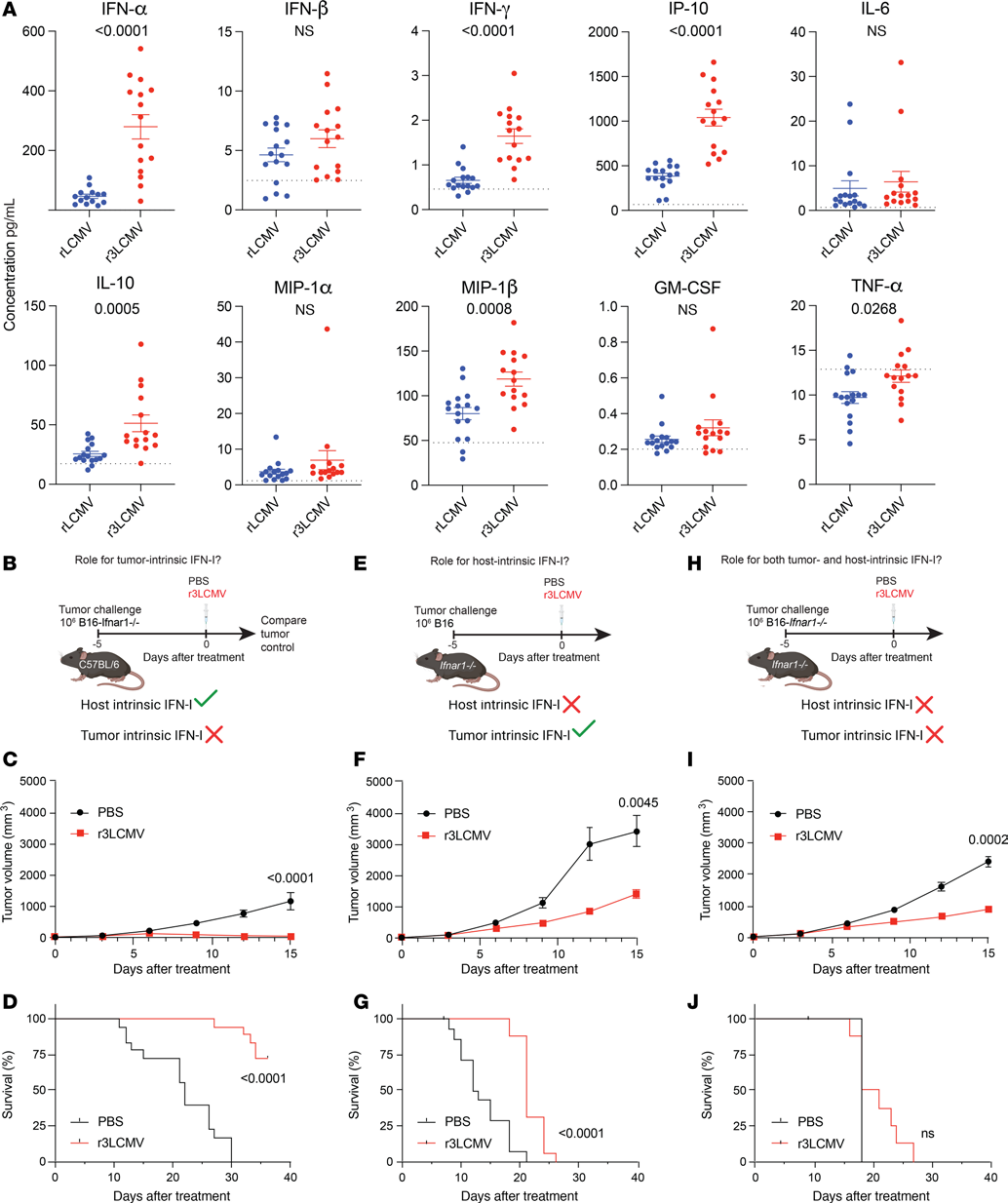
Confirmatory mechanistic studies corroborate a role for IFN-I. (**A**) Cytokine responses at day 1 after treatment. Dotted lines represent naive levels. (**B**–**D**) Effect of r3LCMV vectors on B16-*Ifnar1^–/–^* melanoma. (**B**) Experiment outline for evaluating the role of tumor-intrinsic IFN-I. (**C**) Tumor control. (**D**) Survival. (**E**–**G**) Effect of r3LCMV vectors on *Ifnar1^–/–^* mice. (**E**) Experiment outline for evaluating the role of host-intrinsic IFN-I. (**F**) Tumor control. (**G**) Survival. (**H**–**J**) Effect of r3LCMV vectors on B16-*Ifnar1^–/–^* melanoma in *Ifnar1^–/–^* mice. (**H**) Experiment outline for evaluating the combined role of tumor-intrinsic and host-intrinsic IFN-I. (**I**) Tumor control. (**J**) Survival. Data from **A** are pooled from 3 experiments (one experiment with *n* = 5 per group, another with *n* = 5 per group, and another with *n* = 5–6 per group). Data from **B**–**G** are pooled from 2 experiments (one experiment with *n* = 8–9 per group, and another with *n* = 8–10 per group). Data from **H**–**J** are pooled from 2 experiments (*n* = 4–5 per group). Error bars represent SEM. Indicated *P* values were calculated by the Mann-Whitney test, or log rank test when comparing survival.

We then validated the gene expression data at the protein level. IFN-I and interferon-induced cytokines were highly upregulated in the serum of mice treated with r3LCMV (Figure 6A), consistent with other studies examining cytokine responses with other LCMV vectors (7, 12). IFN-I levels were higher after treatment with the replicating vector (r3LCMV) relative to the non-replicating vector (rLCMV). We also performed mechanistic validation of our scRNA-Seq data. In particular, we evaluated the mechanistic roles of IFN-I by treating tumor-bearing mice with an IFN-I receptor–blocking antagonist (αIFNAR1 antibody, clone MAR1-5A3), which has been used in prior studies to block the IFN-I pathway (12, 19–22). Blockade of the IFN-I pathway significantly blunted the antitumoral efficacy of r3LCMV therapy (Supplemental Figure 6), suggesting that IFN-I could play a role in the antitumoral effect.

Next, we performed 3 series of experiments to determine the tumor-intrinsic versus host-intrinsic roles of IFN-I. In our first model, we challenged mice with B16 melanoma cells lacking IFNAR1 (B16-*Ifnar1^–/–^*) (Figure 6B). These mice lacking IFN-I signaling specifically on tumor cells exhibited potent antitumoral responses and improved survival after r3LCMV treatment, suggesting that tumor-intrinsic IFN-I was dispensable (Figure 6, C and D). In our second model, we challenged *Ifnar1^–/–^* mice with B16 melanoma cells. In this model, in which the host cells could not sense IFN-I, we observed that the antitumoral effect was modest and all mice succumbed within 4 weeks, suggesting that host-intrinsic IFN-I was important (Figure 6, E–G). In our third model, we challenged *Ifnar1^–/–^* mice with B16-*Ifnar1^–/–^* melanoma cells. In this third model, in which both the host and the tumor were unable to sense IFN-I, the antitumoral effect of r3LCMV was dampened and there was no significant improvement in survival following r3LCMV therapy (Figure 6, H–J). These data suggest that host-intrinsic IFN-I signaling is critical for the antitumoral effect of r3LCMV therapy.

### r3LCMV treatment induces antitumoral effects without the need for NK cells, macrophages, or MyD88.

**Figure 7:**
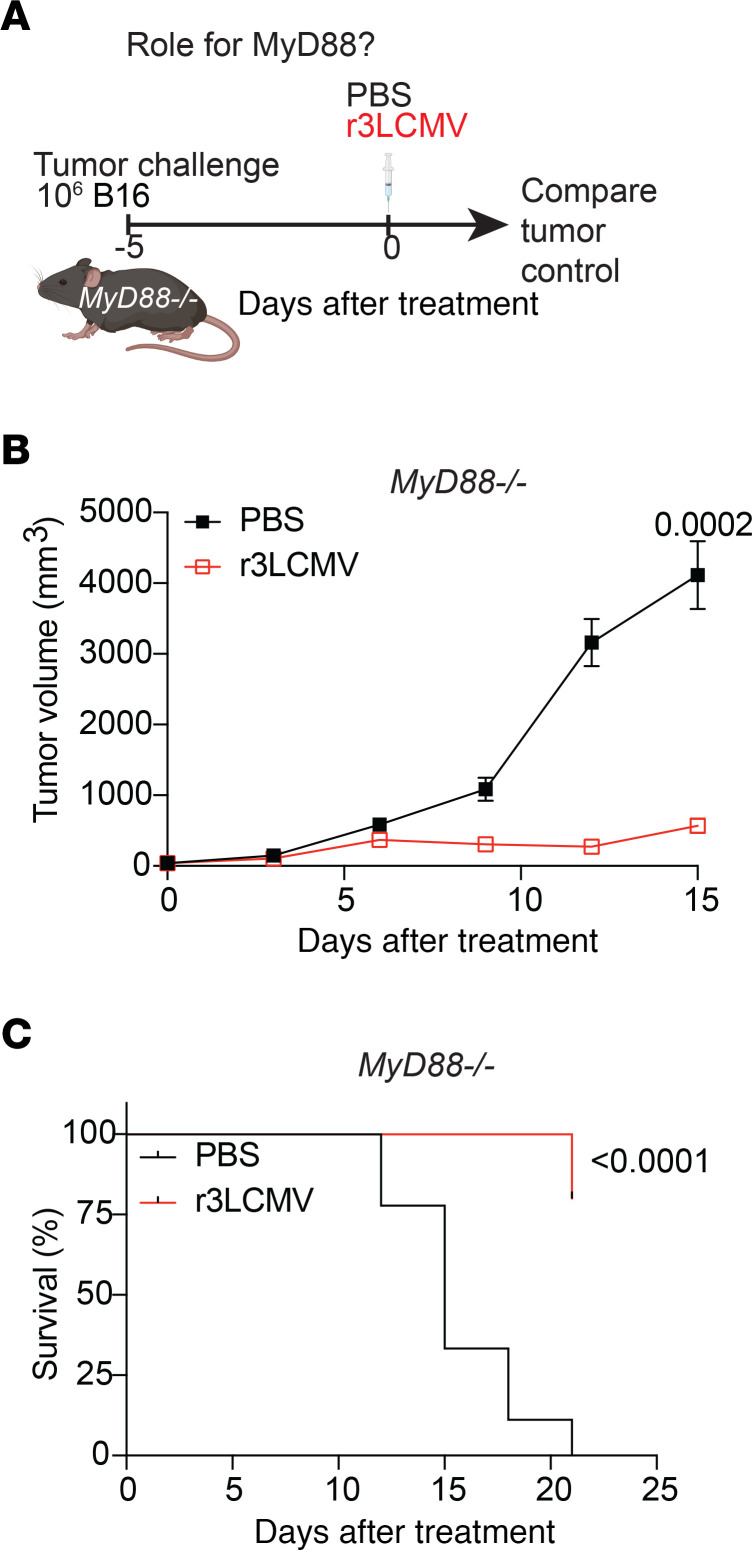
r3LCMV therapy improves tumor control in *MyD88^–/–^* mice. (**A**) Experiment outline. The setup was similar to that in Figure 1, but using *MyD88^–/–^* mice instead of wild-type mice. (**B**) Tumor control. (**C**) Survival. Data are pooled from 2 experiments (*n* = 4–5 per group/experiment). Error bars represent SEM. Indicated *P* values were calculated by the Mann-Whitney test, or log rank test when comparing survival.

Since the scRNA-Seq studies showed enrichment in NK cells and macrophages within the tumor upon r3LCMV treatment, we examined the contribution of these cells in tumor control. We first challenged mice with B16 melanoma tumors and then depleted NK cells continuously with an NK cell–depleting antibody to determine whether the antitumoral effect of r3LCMV is mediated by NK cells. Our results indicate that NK cell depletion did not abrogate tumor control by the r3LCMV treatment (Supplemental Figure 7, A and B). Similarly, continuous depletion of macrophages using clodronate liposomes did not abrogate tumor control after r3LCMV treatment (Supplemental Figure 7, C and D). Altogether, the antitumoral effect of r3LCMV did not require NK cells and macrophages. In addition, we examined whether the antitumoral effects of r3LCMV were dependent on MyD88, an adaptor protein downstream of most Toll-like receptors (TLRs) that is considered to play a central role in innate immune responses. Interestingly, the r3LCMV therapy was still effective in tumor-bearing *MyD88^–/–^* mice, demonstrating that MyD88 was not required for the antitumoral effect (Figure 7).

### r3LCMV induces a long-lasting antitumoral state.

Tumors can recur throughout the lifespan of the host. In our experiments, all control PBS-treated mice succumbed to the B16 melanoma challenge within weeks of tumor challenge, whereas a fraction of r3LCMV-treated mice typically survived. We interrogated whether mice that had cleared tumors (following r3LCMV therapy) developed immune memory to the tumor. Surviving mice that were previously treated with r3LCMV and that had cleared B16 tumors showed enhanced tumor control following a secondary tumor challenge, relative to control naive mice (Supplemental Figure 8), suggesting that r3LCMV induced a memory response to the tumor.

**Figure 8:**
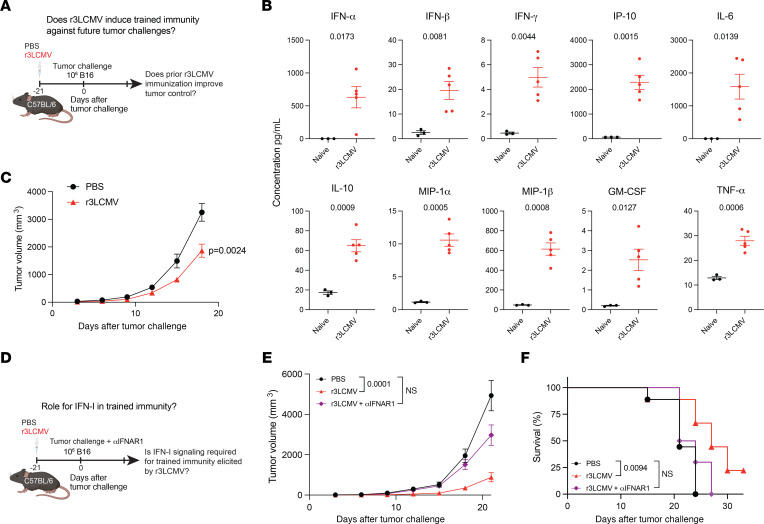
Prior treatment with r3LCMV renders mice more resistant to tumor challenges. We tested whether mice that had previously been injected with r3LCMV were protected upon subsequent tumor challenges. (**A**) Experiment outline to evaluate the effect of prior r3LCMV treatment on subsequent tumor challenges (trained immunity). (**B**) Cytokine responses at day 1 after r3LCMV treatment. (**C**) Tumor control. (**D**) The experiment in **A** was repeated, but mice were treated with IFNAR1-blocking antibodies at the time of tumor challenge (see Methods) to examine the role of IFN-I signaling. (**E**) Tumor control in the context of IFNAR1 blockade. (**F**) Survival in the context of IFNAR1 blockade. Cytokine data from **B** are from 1 experiment with *n* = 5 mice (naive mice are shown as controls); the experiment was repeated with similar results. Data from **C** are from 2 experiments, *n* = 13 per group. Data from **D**–**F** are from 2 experiments (*n* = 4–5 per group). Error bars represent SEM. Indicated *P* values in cytokine plots were calculated by Welch’s *t* test. Indicated *P* values in the tumor volume plots were calculated by the Mann-Whitney test, or by Kruskal-Wallis test with Dunn’s multiple-comparison test when comparing more than 2 groups. Indicated *P* values in the survival plot were calculated by the log rank test.

Until now, all of our r3LCMV treatments had been in tumor-bearing mice to examine its effect as a therapeutic regimen for cancer. But we also performed the “inverse” experiment by first treating mice with r3LCMV and then challenging them with B16 tumors several weeks after (Figure 8A). Treatment of non-tumor-bearing mice with r3LCMV induced proinflammatory cytokines in serum, including IFN-I (Figure 8B). When challenged 3 weeks later with B16 melanoma, these r3LCMV-immune mice exhibited improved tumor control relative to r3LCMV-naive mice (Figure 8C). Since LCMV does not share any epitopes with the B16 melanoma, this observation suggested induction of trained immunity. Trained immunity is a poorly understood process by which prior infections can trigger epigenetic changes in the innate immune system, resulting in antigen-nonspecific immune protection against unrelated antigens (23). Prior studies have suggested a role for IFN-I in promoting trained immunity (24, 25), motivating us to examine its role. We first treated naive mice with r3LCMV, and after 3 weeks, we treated these mice with control antibodies or IFNAR1-blocking antibodies, followed by B16 tumor challenge (Figure 8D). Interestingly, tumor control and survival in r3LCMV-immune mice were severely impaired when the IFN-I pathway was blocked, demonstrating a role for IFN-I (Figure 8, E and F). Overall, we demonstrate that r3LCMV vectors potentiate tumor control when they are administered after or before tumor challenges, suggesting not only therapeutic but also preventive antitumoral effects.

## Discussion

Tumor vaccines based on recombinant viruses expressing a tumor antigen payload or oncolytic viruses that lyse tumor cells have emerged as promising anticancer agents because of their ability to activate innate and adaptive immune responses, and directly kill cancer cells. For example, enteric cytopathic human orphan virus type 7 (ECHO-7) is currently being used for melanoma owing to its ability to lyse tumor cells (26). In addition, an adenovirus-based vector is used for head and neck cancer; and a herpes simplex virus–based vector is used for recurrent melanoma (2, 27). Less work has been done with nonlytic viruses that do not express any tumor antigen payload and do not directly kill tumor cells. Recently, LCMV vectors encoding HPV16 antigens started clinical trials for the treatment of HPV-related cancers. LCMV is a non-lytic arenavirus in clinical development as a vaccine vector to deliver tumor antigens to the immune system. LCMV does not directly lyse tumor cells, but it induces potent innate and adaptive immune responses that can eliminate infected cells. LCMV is also relatively proficient at evading antibody responses, allowing its reutilization as a vaccine vector in a seropositive host (8, 28).

Prior research has shown that LCMV vectors can outperform protective efficacy elicited by other viral vector platforms, including Ad5 and poxvirus vectors (7, 29). When tumor-bearing mice are immunized with LCMV vectors containing the tumor antigen, they demonstrate stronger antitumor control relative to mice immunized with Ad5 or poxvirus vectors containing the same tumor antigen. However, ongoing clinical trials with LCMV vectors engineered to express tumor antigens have not assessed the potential contribution of bystander (tumor-nonspecific) responses or whether immune activation by the viral vector itself can modulate tumor control.

Prior studies using models of therapeutic vaccination have shown that tumor-specific T cells play a critical antitumoral role in LCMV vector–based cancer therapy (7, 11), but it remains unclear whether LCMV vectors that do not express any tumor antigen can also exert antitumoral responses. Historically, the use of the viral vectors as cancer vaccines requires knowledge of the specific neoantigens or tumor-associated antigens encoded by the tumor, which may vary between different patients and tumor types. In our study, however, we used a “generic” r3LCMV platform that does not encode any tumor antigen. Since the r3LCMV vector and the tumor do not share any antigenic sequence, the antitumoral effect that we report could be considered bystander. Earlier studies by Lang and others showed that infection of tumor-bearing mice with chronic virulent strains of LCMV can improve tumor control, suggesting also a bystander antitumoral effect (30–33). However, safety concerns with the use of live LCMV have deemed this approach hard to translate to humans. A prior clinical trial used live LCMV in cancer patients, but these patients died with evidence of multi-organ LCMV infection upon necropsy (34). These patients were in the late stages of lymphoma, and it was unclear whether they died for that reason; one patient showed bacterial infection at the time of death, so it was unclear whether death was caused by the chronic LCMV infection or the bacterial infection. Chronic LCMV infection can also render the host more susceptible to other diseases as a result of the generalized immunosuppression associated with persistent viral infection (35). Considering these findings, attenuated replicating LCMV vectors represent a safer clinical approach, given their high immunogenicity despite their limited ability to replicate. We report their safety and efficacy even in *Rag1^–/–^* mice.

In our study, we used an attenuated r3LCMV vector that replicates at substantially lower levels than the parental virus but is still able to trigger a robust innate and adaptive immune response. Very low levels of systemic virus can be detected 72 hours after infection with attenuated r3LCMV, with mice showing only a very transient viremia near the limit of detection (<5 PFU/mL) with no weight loss or signs of disease (12). Our studies suggest that T cells, B cells, NK cells, and macrophages (as well as other phagocytes that can be depleted by clodronate liposomes, like monocytes, dendritic cells, and neutrophils; ref. 36) are not absolutely necessary for the antitumoral effect of r3LCMV. However, we found that activation of virus-specific CD8^+^ T cells can facilitate tumor control by r3LCMV, as shown by our P14 adoptive transfer studies. We also demonstrated a critical role for host-intrinsic IFN-I signaling. Future studies will examine the contribution of IFN-I signaling on more specific immune subsets, such as dendritic cells and monocytes. In addition, we demonstrated that *MyD88^–/–^* mice respond to the r3LCMV therapy, which suggests that this major component of innate immunity is also dispensable for the antitumoral effect. It is possible that in the absence of MyD88, other adaptor molecules may compensate for the defects in innate immunity. Overall, the antitumoral effects of r3LCMV seem to engage multiple immune mechanisms, besides adaptive and innate (MyD88-dependent) immunity. Although unlikely, it is important to consider the potential for genetic recombination for) r3LCMV vectors, and future studies should examine safety with these vectors more rigorously. Our data also suggest that r3LCMV induces trained immunity to the tumor, since prior treatment with r3LCMV rendered the mice significantly more resistant to subsequent tumor challenges. In this context, IFN-I seemed to be mechanistically important. In summary, we demonstrate that attenuated r3LCMV vectors exert antitumoral effects in great part via IFN-I and that they are effective even in immunodeficient hosts without adaptive immunity. These studies are important for the development of LCMV-based therapies for cancer and for improving the mechanistic understanding of how nonlytic viral vectors modulate tumor immunity.

### Limitations of the study.

Absence of host-intrinsic IFN-I signaling limits the antitumor efficacy of r3LCMV, yet mice devoid of host-intrinsic IFN-I signaling show partial tumor control upon r3LCMV treatment, suggesting that other immune pathways may contribute to the antitumoral effects. Future studies will examine the contribution of other innate immune pathways that do not depend on MyD88 and examine more thoroughly how r3LCMV mediates trained immunity to tumors.

## Methods

### Sex as a biological variable.

Our study examined male and female animals, and similar findings are reported for both sexes.

### Mice, tumor challenges, and LCMV vector treatments.

Experiments were performed with 6- to 8-week-old wild-type mice (half males and half females) from The Jackson Laboratory (C57BL/6, stock 000664; BALB/c, stock 000651; *Ifnar1^–/–^*, stock 028288; *Rag1^–/–^*, stock 002216; *MyD88^–/–^* mice, stock 009088). Mice were challenged subcutaneously with 10^6^ B16 melanoma cells, MC38 colon adenocarcinoma cells, and CT26 colon carcinoma cells, and r3LCMV treatments started at day 5. Tumor volume was calculated as length × width × width × 1/2. Mouse challenges were performed at Northwestern University following biosafety level 2 guidelines with approval by the Institutional Animal Care and Use Committee.

### Cells and viruses.

We used the murine melanoma cell line B16 (gift from Chyung-Ru Wang, Northwestern University, Chicago, Illinois, USA), the B16-OVA melanoma cell line (gift from Jennifer Wu, Northwestern University), B16-*B2m^–/–^* cells (gift from Omar Abdel-Wahab, Memorial Sloan Kettering Cancer Center, New York, New York, USA), B16- *Ifnar1^–/–^* KO and B16-*Ifngr1^–/–^* cells (InvivoGen), and MC38 (ATCC) and CT26 (ATCC) cells. The tumor cells were cultured in DMEM (GIBCO, catalog 11965-092) with 10% FBS (Sigma-Aldrich, catalog F0926), 1% l-glutamine (GIBCO, catalog 25030-081), and 1% penicillin and streptomycin (GIBCO, catalog 15140-122) in a 37°C, 5% CO_2_ incubator. BHK-21 cells (ATCC, catalog CCL-10) were used for production of LCMV, VSV, and YFV-17D. Vero E6 cells (ATCC, catalog CRL-1586) were used for titration of r3LCMV, VSV, and YFV-17D. BHK-21 and Vero E6 cells were cultured in MEM (ATCC, catalog 30-2003) with 10% FBS, 1% l-glutamine, and 1% penicillin/streptomycin in a 37°C, 5% CO_2_ incubator. Non-replicating (rLCMV) vectors expressing GFP (used in Figure 2, Figure 6A, and Supplemental Figure 1) were a gift from Hookipa Pharma Inc. (Vienna, Austria). For the rest of the experiments, we used replicating (r3LCMV) vectors expressing GFP, which were made using DNA plasmids from Juan Carlos De La Torre (Scripps Research Institute, La Jolla, California, USA). The LCMV strain lacking the GP33-41 epitope (GP35V→A escape mutation, which cannot be recognized by P14 cells) was derived from a prior study (17). This LCMV variant was used to examine the role of virus-specific CD8^+^ T cell activation in tumor control.

### Adoptive cell transfer.

CD8^+^ T cells were purified from spleens of transgenic P14 mice, using a negative selection isolation kit (STEMCELL Technologies), and purity was confirmed to be greater than 97%. CD8^+^ T cells (5 × 10^6^) were injected into a mouse intravenously, 1 day before viral infection.

### Antibody treatments and cell depletions.

All antibodies for in vivo treatments were purchased from Leinco Technologies and were diluted in sterile PBS and injected intraperitoneally. PD-L1–blocking antibodies (clone 10F.9G2) were administered at 200 μg, every 3 days, 5 times, as previously shown (37). B7.1- and B7.2-blocking antibodies (clones 16-10A1 and GL-1, respectively) were administered at 200 μg each, every 3 days. IFNAR1-blocking antibodies (clone MAR1-5A3) were administered at 200 μg, every 3 days, 5 times. This MAR1-5A3 antibody binds to IFN-α/β receptor subunit 1 (clone IFNAR1) and blocks binding to IFN-α and IFN-β, abrogating the induction of ISGs in vivo (19, 20, 22, 38, 39). NK cell–depleting antibodies (clone NK1.1 PK136) were administered at 500 μg, every 2 days, 5 times. CD4^+^ T cell–depleting antibodies (clone GK1.5) were administered at 200 μg, 2 times, and CD8^+^ T cell–depleting antibodies (clone 2.43) were administered at 200 μg, every 3 days, 5 times (starting on the day of r3LCMV treatment). Diphtheria toxin (Sigma-Aldrich) was administered at 1 μg i.p. (diluted in PBS), on days 0, 1, 4, 7, and 10 of r3LCMV therapy. This dose was similar to those in prior studies using FoxP3-DTR knockin mice on a C57BL/6 background (14, 40). Clodronate liposomes (Encapsula NanoSciences, SKU CLD-8909) were administered at 200 μg every 3 days, 4 times.

### Quantification of viral titers.

Viral titers were quantified as described previously (41). In brief, 5 × 10^5^ Vero E6 cells were plated onto each well in 6-well plates, and after 24–48 hours when they reached approximately 95% confluence, the media were removed and 200 μL of serial dilutions (of viral stock or tissue homogenates) were added dropwise on top of the monolayer of the cells. Plates were rocked every 10 minutes in a 37°C, 5% CO_2_ incubator for 1 hour. Two hundred microliters of medium was aspirated out, and the monolayers were gently overlaid with a 1:1 mixture of 2× 199 medium (20% FBS, 2% penicillin/streptomycin, 2% l-glutamine) and 1% agarose at 37°C. After 4 days, a second overlay was added, consisting of a 1:1 solution of 2× 199 medium, 1% agarose, and 1:50 of neutral red. Overlay was removed on day 5, and plaques were counted using a conventional light microscope.

### Flow cytometry.

MHC class I monomers (K^b^SIINFEKL or D^b^GP33) were used for detecting virus-specific CD8^+^ T cells, and were obtained from the NIH tetramer facility located at Emory University (Atlanta, Georgia, USA). MHC I monomers were tetramerized in-house. Single-cell suspensions were stained with live/dead fixable dead cell stain (APC-Cy7, Invitrogen, catalog L34976A), anti–mouse CD8α (clone 53-6.7, PerCP-Cy5.5, BD Pharmingen, catalog 551162; clone 53-6.7, FITC, BD Pharmingen, catalog 553031; clone 53-6.7, APC, eBioscience, catalog 17-0081-83), anti–mouse CD4 (clone RM4-5, PE-Cy7, eBioscience, catalog 25-0042-82; clone RM4-5, Pacific blue, eBioscience, catalog 57-0042- 82), anti–mouse CD44 (clone IM7, FITC, BD Pharmingen, catalog 553133; clone IM7, Pacific blue, BioLegend, catalog 103020), anti–mouse CD80 (clone 16-10A1, FITC, BD Pharmingen, catalog 553768), anti–mouse CD86 (clone GL1, PE, BD Pharmingen, catalog 561963), anti-IFNAR1 (clone MAR1-5A3, PE, BioLegend, catalog 127312), anti–mouse CD11b (clone M1/70, Alexa Fluor 700, BioLegend, catalog 101222), anti–mouse CD11c (clone N418, PerCP-Cy5.5, BioLegend, catalog 117328; clone N418, PE-Cy7, BioLegend, catalog 117318), anti–mouse CD90.1 (Thy1.1) (clone HIS51, eFluor 450, eBioscience, catalog 48-0900-82; clone HIS51, PE-Cy7, eBioscience, catalog 25-0900-82), anti–mouse CD90.2 (Thy1.2) (clone 53-2.1, APC, eBioscience, catalog 17-0902-82), anti–mouse CD45.1 (clone A20, PE-Cy7, BioLegend, catalog 110730; clone A20, FITC, BD Pharmingen, catalog 553775), anti–mouse CD45.2 (clone 104, PE, BioLegend, catalog 109808; clone 104, FITC, BD Pharmingen, catalog 553772), anti–mouse TCR Va2 (clone B20.1, PE, BD Pharmingen, catalog 553289), anti–mouse CD279 (PD-1) (clone RMP1-30, PE, BioLegend, catalog 109104; clone RMP1-30, PE-Cy7, BioLegend, catalog 109110; clone RMP1-30, FITC, eBioscience, catalog 11-9981-85), anti–mouse CD274 (B7-H1, PD-L1) (clone 10F.9G2, PE, BioLegend, catalog 124308), anti–mouse FoxP3 (clone FJK-16s, APC, eBioscience, catalog 17-5773-82), anti–mouse CD25 (clone 3C7, PerCP-Cy5.5, BioLegend, catalog 101911), anti–mouse B220 (clone RA3-6B2, PerCP-Cy5.5, BioLegend, catalog 103236), anti–mouse CD3 (clone 17A2, Pacific blue, BioLegend, catalog 100214; clone 17A2, biotin, BioLegend, catalog 100244), anti–mouse Ly-6G (clone RB6-8c5, biotin, eBioscience, catalog 13-5931-85), anti–mouse NK1.1 (clone PK136, biotin, eBioscience, catalog 13-5941-85; clone PK136, PE, BD Pharmingen, catalog 553165), anti–mouse CD19 (clone eBio1D3, biotin, eBioscience, catalog 13-0193-82), SA-BV421 (BioLegend, catalog 405225), SA-APC (Invitrogen, catalog S868), and SA-PE (BioLegend, catalog 405204). Flow cytometry samples were acquired with a Becton Dickinson Canto II or an LSRII and analyzed using FlowJo v10 (Tree Star).

### Tumor sectioning and immunofluorescence.

Tumors were fixed in PLP fixative solution (paraformaldehyde [Thermo Scientific, 28908]; L-Lysine [Sigma, L5626]; sodium m-Periodate [Sigma, S1878]) for 24 hours at 4°C. The tumor samples were washed with PBS and cryoprotected for 24 hours at 4°C in a sucrose/PBS dilution. The fixed tissue samples were frozen in OCT compound on dry ice. Once the samples were frozen, they were kept in a –80°C freezer until sectioning. The tissue samples were sectioned using a cryomicrotome with 10 μm thickness. The frozen sections were washed with PBS 2 times for 5 minutes each time and rinsed in 0.05% PBS-T. The slides were incubated with the blocking solution (PBS plus 1% BSA plus 5% goat serum) for 10 minutes. The slides were stained with the primary and the secondary antibodies in the blocking solution for 2 hours and 1 hour, respectively. VL4 antibody (Bio X Cell) was used to detect LCMV antigen. After the primary and the secondary antibody staining, the slides were washed 2 times with PBS-T. The slides were washed with water and mounted with Vector AntiFade mounting medium. Slides were imaged at the Center for Advanced Microscopy Cell Imaging Facility and Nikon Imaging Center at Northwestern University.

### Multiplex cytokine/chemokine assay.

The mouse peripheral blood samples were collected in 1.5 mL tubes 24 hours after infection of LCMV. The blood samples were centrifuged at 21,130 rcf at 4°C to separate the serum samples. The serum samples were collected and frozen at –80°C until its use. Multiplex cytokine/chemokine kit was purchased from Mesoscale Diagnostics LLC.

### LCMV-specific ELISA.

Binding antibody titers were quantified by ELISA as described previously (12, 41–46), using LCMV GP as coating antigen. Briefly, 96-well, flat-bottom MaxiSorp plates (Thermo Fisher Scientific) were coated with 1 μg/mL of GP for 48 hours at 4°C. Plates were washed 3 times with wash buffer (PBS plus 0.05% Tween-20). Blocking was performed with blocking solution (200 μL PBS plus 0.05% Tween-20 plus 2% BSA) for 4 hours at room temperature. Six microliters of plasma samples were added to 144 μL of blocking solution in the first column of the plate, 3-fold serial dilutions were prepared for each sample, and plates were incubated for 1 hour at room temperature. Plates were washed 3 times with wash buffer. Goat anti-mouse IgG antibody tagged with streptavidin-HRP (Southern Biotech, 7105-05) was diluted 1:400 in blocking buffer and incubated for 1 hour at room temperature. After washing of plates 3 times with wash buffer, 100 μL/well SureBlue Substrate (SeraCare) was added for 1 minute. The reaction was stopped using 100 μL/well KPL TMB Stop Solution (SeraCare). Absorbance was measured at 450 nm using SpectraMax Plus 384 (Molecular Devices).

### Single-cell RNA sequencing.

Five different mice treated with r3LCMV and 5 different mice treated with vehicle (PBS) were enriched for CD45^+^ cells and pooled for single-cell sequencing, separately. Single-cell libraries were generated using 10x Genomics 3′ kits. Cell Ranger (version 6.1.2) was used to demultiplex raw base call files (BCL) to FASTQ files and align reads to the mouse genome (Ensembl version GRCm39 version 110) supplemented with LCMV genome (GenBank accession NC_004291.1 and NC_004294.1). For counting, Cell Ranger was run with the option to include reads spanning intron regions of genes during counting; all remaining default options were used. Count matrices were further analyzed in R (version 4.6.2), Bioconductor (version 3.17), and the R package Seurat (version 4.3.0.1). The R package SingleR (version 2.2.0) with the Immunological Genome Project (ImmGen) reference was used to annotate the subset of each cell. Differential expression was performed by fitting of a negative binomial generalized linear model to gene expression and a likelihood-ratio test for statistical testing. Benjamini-Hochberg adjustment was used to correct for multiple testing, and cutoff of 5% false-positive was considered significant. scRNA-Seq was performed at the Northwestern University NUSeq core.

### Statistics.

Statistical analyses are indicated in the figure legends. Statistical significance was established at *P* ≤ 0.05. In the figures showing tumor control over time, the *P* values were calculated based on the tumor sizes at the last time point shown. Data were analyzed using GraphPad Prism.

### Study approval.

Mouse studies were performed at Northwestern University following biosafety level 2 guidelines with approval of the Institutional Animal Care and Use Committee under protocol IS00003324.

### Data availability.

scRNA-Seq data were uploaded to the NCBI’s Gene Expression Omnibus database (GEO GSE255499; https://www.ncbi.nlm.nih.gov/geo/query/acc.cgi). Other data are available upon request. Supporting data values associated with the main article and supplemental material are included in the Supporting Data Values file.

## Author contributions

YRC and BA performed most of the mouse tumor challenge and immunogenicity experiments. Authorship order was decided mutually by these two co–first authors. TD and NI helped to perform some of the mouse experiments. SF analyzed the gene expression data. PPM designed the experiments and secured funding. PPM wrote the paper with feedback from all authors. The gene expression analysis in this research was supported in part through the computational resources and staff contributions provided by the Genomics Compute Cluster, which is jointly supported by the Feinberg School of Medicine, the Center for Genetic Medicine, the Feinberg’s Department of Biochemistry and Molecular Genetics, the Office of the Provost, the Office for Research, and Northwestern Information Technology.

## Supplementary Material

Supplemental data

Supporting data values

## Version 1 06/11/2024

In-Press Preview

## Version 2 08/01/2024

Electronic publication
