# Supplementary material for: An attenuated lymphocytic choriomeningitis virus vector enhances tumor control in mice partly via IFN-I
Source: J Clin Invest. 2024 Jun 11;134(15):e178945. doi: 10.1172/JCI178945 (PMC11290963; doi:10.1172/JCI178945)
Supplement: Supplemental data [file jci-134-178945-s093.pdf]

Supplementary Material

An attenuated lymphocytic choriomeningitis virus vector enhances tumor control in mice partly via IFN-I

Young Rock ChungΨ[1], Bakare AwakoaiyeΨ[1], Tanushree Dangi[1], Nahid Irani[1], Slim Fourati[2], Pablo Penaloza-MacMaster*[1]

[1]Department of Microbiology-Immunology, Feinberg School of Medicine, Northwestern University, Chicago, IL 60611, USA. [2]Department of Medicine, Division of Allergy and Immunology, Feinberg School of Medicine and Center for Human Immunobiology, Northwestern University, Chicago, IL 60611, USA.

Ψ **Equal contribution**

*** Correspondence and Lead contact:**

Pablo Penaloza-MacMaster (ppm@northwestern.edu)

303 E Chicago Ave, Tarry 6-729, Chicago, IL, 60611, U.S.A.

Phone: 312-503-0357

Supplemental Figures:

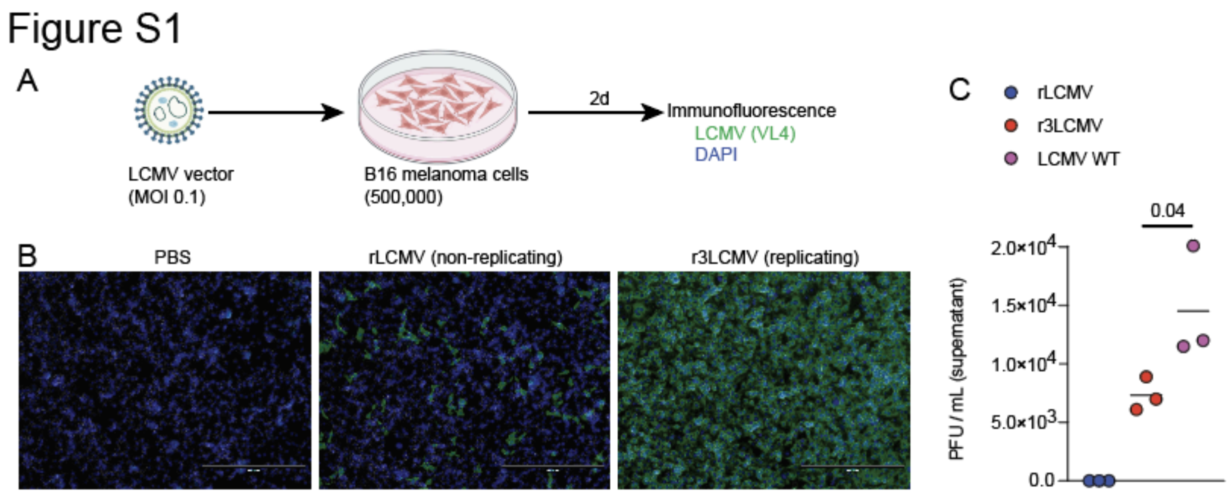

Supplemental Figure 1. r3LCMV replicates in B16 melanoma cells. (**A**) Experiment outline for detecting viral antigen after in vitro infection of B16 melanoma cells with non-replicating (rLCMV) or replicating (r3LCMV) vectors. (**B**) Representative immunofluorescence staining in B16 monolayers at day 4 post-infection. In this experiment, we detected substantially more viral antigen in B16 monolayers that were infected with replicating (r3LCMV), relative to non-replicating (rLCMV) vector. Scale bars represent 400 μm. (**C**) Viral loads from B16 supernatants harvested at day 2 post-infection. Viral load quantification was performed by plaque assays on Vero cell monolayers. No infectious virus was detected in supernatants of rLCMV-infected B16 cells. LCMV wild-type (Cl-13) was used as control. Data are from one experiment with 3 replicates. Experiment was repeated with similar results. Indicated P value was calculated by one-way ANOVA (Holm-Šídák's multiple comparisons test).

Figure S2

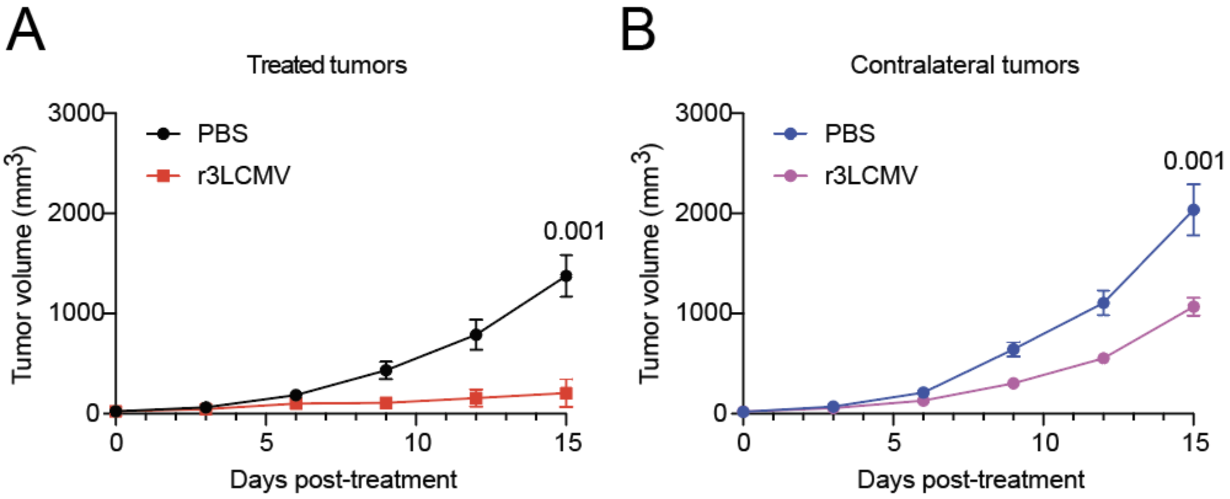

Supplemental Figure 2. Attenuated r3LCMV induces abscopal effect. B16 tumor-bearing mice were treated intratumorally with $2x10^5$ FFU of r3LCMV on the left tumor only, five days after bilateral tumor challenge. In this model, both flanks were injected with B16 melanoma cells, but only one flank was treated with r3LCMV. (**A**) Tumor control on the treated tumor (left side). (**B**) Tumor control on the contralateral untreated tumor (right side). Although most of the therapeutic effect was observed on the treated tumor, there was partial regression of the distal contralateral tumor, suggesting abscopal effect. Data are from 2 experiments with a total of n=9-10 per group. Error bar represents SEM. Indicated P values were calculated by the Mann–Whitney test.

Figure S3

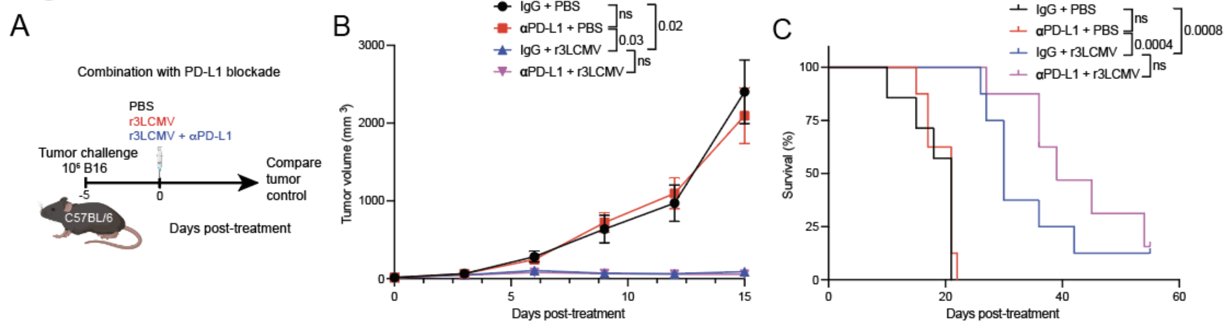

Supplemental Figure 3. Effect of combined PD-L1 blockade and r3LCMV therapy. (**A**) Experiment outline for evaluating whether PD-L1 blockade improves r3LCMV therapy. PD-L1 blocking antibodies (clone 10F.9G2) were administered intraperitoneally at 200 μg, every three days, five times, starting on the day of r3LCMV treatment. (**B**)Tumor control. (**C**) Survival. Data are from 1 experiment (n=8 per group). Error bar represents SEM. Indicated P values were calculated by the Kruskal-Wallis test and Dunn's multiple comparison test, or log rank test when comparing survival.

Figure S4

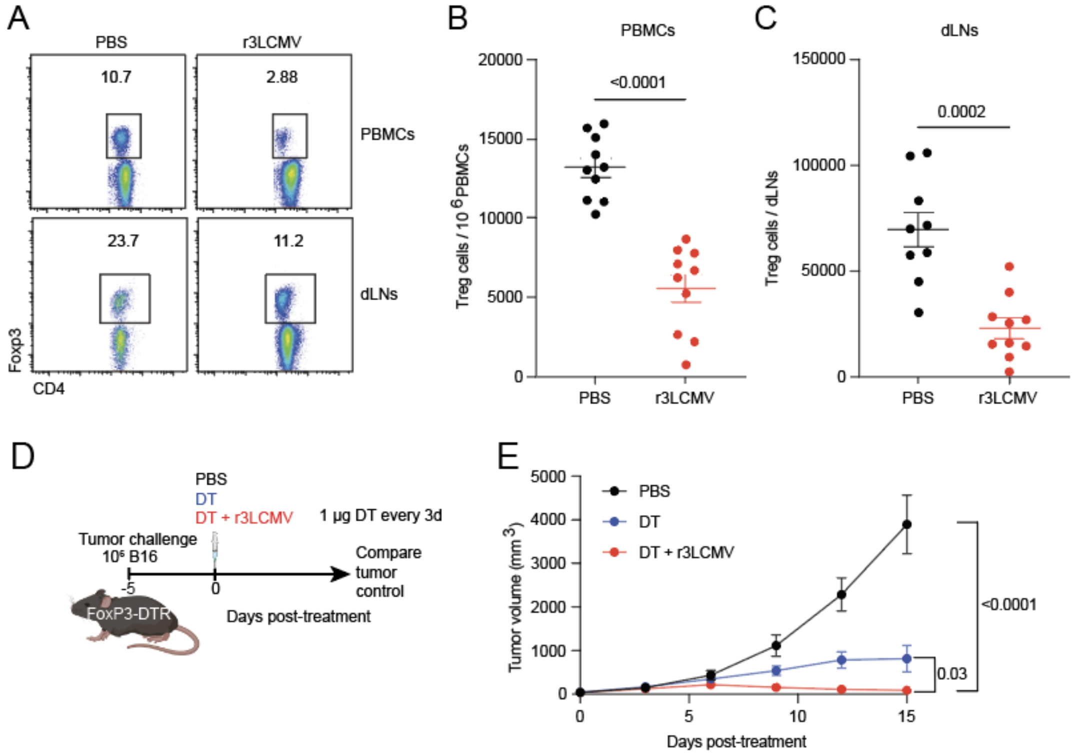

Supplemental Figure 4. r3LCMV therapy results in a decline in Tregs. (**A**) Representative FACS plots showing Treg cell responses (gated on live CD4 T cells). (**B**) Summary of Treg cell responses in PBMCs. (**C**) Summary of Treg cell responses in tumor-draining lymph nodes. Data from PBMCs are from day 7 post-treatment, and data from tumor draining lymph nodes are from day 8 post-treatment. (**D**) FoxP3-DTR mice were challenged with B16 melanoma tumors, similar to Figure 1. After 5 days post-challenge, they were treated with diphtheria toxin (DT), with or without r3LCMV. DT was administered intraperitoneally at 1 μg on days 0, 1, 4, 7, and 10 of r3LCMV therapy (see Materials and Methods for more information). (**E**) Tumor control. Data are pooled from 2 experiments (one experiment with n=3-6 per group and another with n=7-8 per group). Error bar represents SEM. Indicated P values were calculated by the Mann–Whitney test in panels B-C, and the Kruskal-Wallis (Dunn's multiple comparisons) test in panel E.

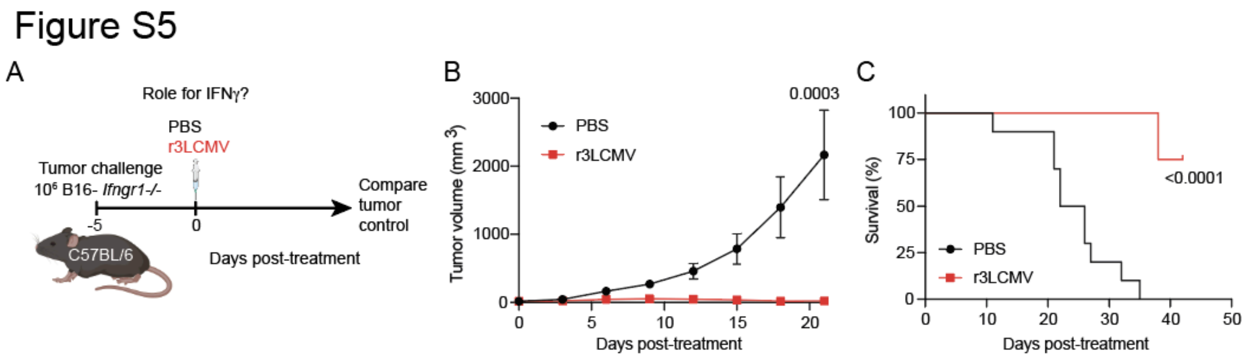

Supplemental Figure 5. Tumor-intrinsic IFNγ signaling is not required for the antitumoral effect of r3LCMV. We tested the effect of r3LCMV vectors on B16 *Ifngr1*-/- melanoma. This tumor cannot sense IFNγ due to lack of its receptor. (**A**) Experiment outline for evaluating the role of tumor-intrinsic IFNγ signaling. Experiment was similar to Figure 1, except that B16 *Ifngr1*-/- melanoma cells were used for challenges. (**B**) Tumor control. (**C**) Survival. Data are from 1 experiment (n=8-9 per group). Error bar represents SEM. Indicated P values were calculated by the Mann–Whitney test, or log rank test when comparing survival.

Figure S6

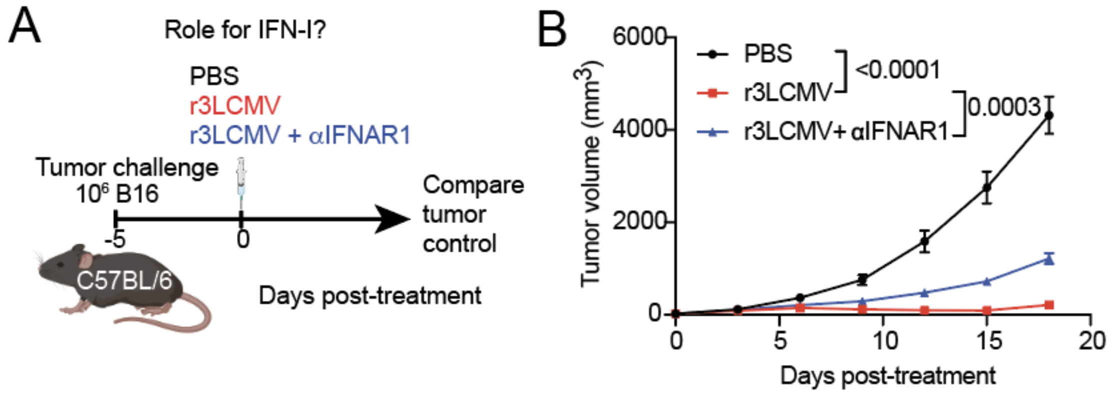

Supplemental Figure 6. IFN-I signaling is partially required for the antitumoral effect of r3LCMV. We tested the effect of IFNAR1 blockade on r3LCMV therapy. (**A**) Experiment outline for evaluating the role of IFN-I signaling. IFNAR1 blocking antibodies (clone MAR1-5A3) were administered intraperitoneally at 200 μg, every three days, five times, starting on the day of r3LCMV treatment. (**B**) Tumor control. Data are pooled from 3 experiments (one experiment with n=6-7 per group, another with n=5, and another with n=9-10 per group). Error bar represents SEM. Indicated P values were calculated using the Kruskal-Wallis test and Dunn's multiple comparisons test.

Figure S7

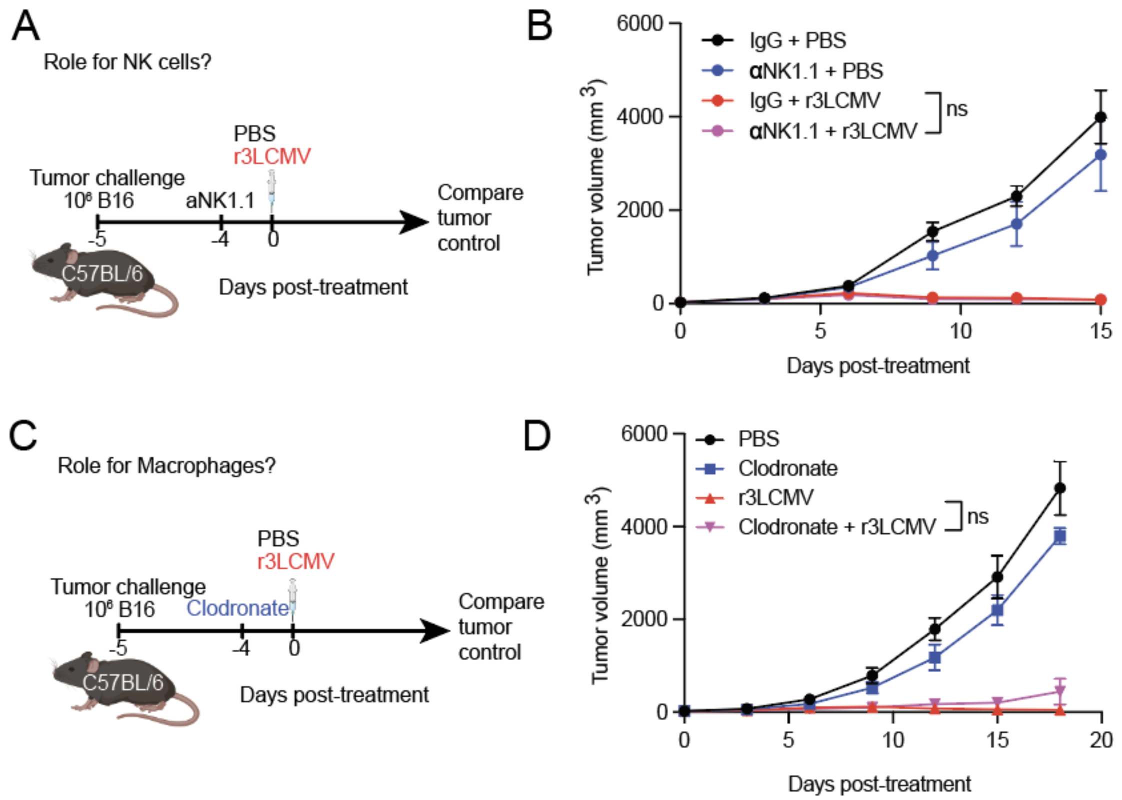

Supplemental Figure 7. NK cells and macrophages are not required for the antitumoral effect of r3LCMV. (**A**) Experiment outline for examining the role of NK cells. NK cell depleting antibodies (clone PK136) were administered at 500 μg, every 2 days, five times (see Materials and Methods). (**B**) Tumor control after NK depletion. (**C**) Experiment outline for examining the role of macrophages. Clodronate liposomes were administered at 200 μg every 3 days, four times (see Materials and Methods for more information). (**D**) Tumor control after macrophage depletion. Data in panel B are from 1 experiment (n=5 per group); Data in panel D are from 1 experiment (n=4-5 per group). Error bar represents SEM. Indicated P values were calculated using the Kruskal-Wallis test and Dunn's multiple comparisons test.

Figure S8

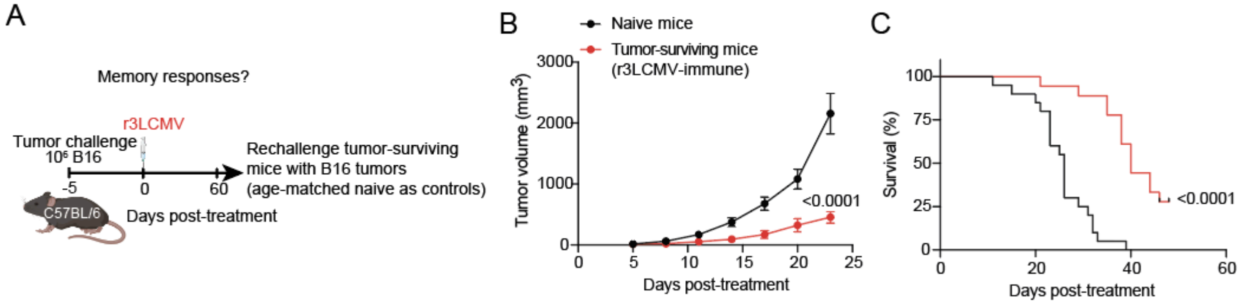

Supplemental Figure 8. Immune memory after treatment of tumor-bearing mice with r3LCMV. We tested whether mice that cleared B16 tumors after r3LCMV therapy were protected upon subsequent tumor challenges. (**A**) Experiment outline to evaluate immune memory to the tumor. Mice that had completely cleared B16 tumors after r3LCMV were re-challenged with B16 tumors to examine anamnestic immune protection. All the mice that received PBS treatment during the initial tumor challenge died, so we used naïve age-matched mice as controls for the tumor-rechallenge experiment. (**B**) Tumor control after tumor-rechallenge. (**C**) Survival. Naïve age-matched mice were used as controls. Data are from 2 experiments, n=9-10 per group. Error bar represents SEM. Indicated P values were calculated by the Mann–Whitney test, or log rank test when comparing survival.
